# microRNA-371a-3p as informative biomarker for the follow-up of testicular germ cell cancer patients

**DOI:** 10.1007/s13402-017-0333-9

**Published:** 2017-06-13

**Authors:** Ton van Agthoven, Wil M. H. Eijkenboom, Leendert H. J. Looijenga

**Affiliations:** Department of Pathology, Laboratory for Experimental Patho-Oncology, Erasmus MC Cancer Institute, Be-432A, P.O. Box 2040, 3000 CA Rotterdam, NL The Netherlands

**Keywords:** Testicular germ cell cancer, microRNA, Serum biomarkers, B-HCG, AFP

## Abstract

**Purpose:**

α-fetoprotein (AFP) and human chorionic gonadotropin subunit beta (B-HCG) are informative serum biomarkers for the primary diagnosis and follow-up of testicular germ cell cancer (TGCC) patients. About 20% of TGCC patients with a non-seminoma (NS) and about 80% with a seminoma (SE) are, however, negative for these biomarkers. Embryonic stem cell microRNAs (miRs) may serve as promising alternative serum biomarkers. Here we investigated a retrospective series of serum samples from selected TGCC patients who developed a relapse in time to test the possible additional value of the serum-based ampTSmiR test compared to the conventional serum-based protein biomarkers for follow-up.

**Methods:**

We investigated 261 retrospective serum samples of six selected fully evaluated TGCC patients with a proven relapse using the ampTSmiR test for miR-371a-3p, miR-373-3p, and miR-367-3p and compared the results to those of the conventional protein biomarkers.

**Results:**

At primary diagnosis, elevated serum B-HCG, AFP and LDH levels were found to be informative in 4/6, 3/6 and 3/6 patients, respectively. At primary diagnosis the levels of miR-371a-3p and miR-373-3p were elevated in 4/4, and miR-367-3p in 3/4 patients. For two cases no starting serum sample was available for retrospective miR analysis. Residual disease (overlooked by histopathological examination) was detected in one case by miR-371a-3p only. The miR-371a-3p level was increased in one patient two months before detection of an intracranial metastasis. B-HCG was informative in 3/4 and the ampTSmiR test in 4/4 patients with a relapse or residual disease. None of the biomarkers were informative for the detection of residual mature teratoma.

**Conclusions:**

The ampTSmiR test is more sensitive than the conventional TGCC protein biomarkers for the detection of residual disease and relapse, excluding mature teratoma.

**Electronic supplementary material:**

The online version of this article (doi:10.1007/s13402-017-0333-9) contains supplementary material, which is available to authorized users.

## Introduction

Although germ cell cancer (GCC) is rare in the general population, it represents the most frequent malignancy in young Caucasian males, predominantly of the testis, accounting for 60% of all malignancies in these males between 20 and 40 years of age [1]. Its incidence is increasing [2] and long-term effects of systemic treatment are evident [3]. Therefore, efforts are being made to diagnose this disease at an early stage. Clinically and histologically, TGCC are divided into two main subtypes, seminoma (SE) and non-seminoma (NS), both derived from pre-invasive germ cell neoplasia in situ (GCNIS) which, in turn, originates from a transformed primordial germ cell or gonocyte [4, 5]. NS can be heterogeneous, consisting of the histologic variants embryonal carcinoma (EC), yolk sac tumor (YST), choriocarcinoma (CH) and teratoma (TE) [1, 4, 6–8]. Although the 5-year survival rate of TGCC patients exceeds 96%, patients have a long term risk of developing a second primary TGCC or of progression of the disease [9]. Therefore, they undergo intensive and long-term follow-up after initial diagnosis, including extensive physical examination and monitoring of the levels of the conventional serum protein markers B-HCG, AFP and to a lesser extent LDH, due to its low specificity [10]. Serum biomarker positivity relates to the histology of the primary (and possibly metastasized) tumor. As such, B-HCG is predominantly related to the presence of a CH component and AFP to a YST component, whereas they show little sensitivity for the detection of SE and the NS stem cell component EC. This explains why a substantial percentage of TGCC patients, predominantly without CH and YST components, can be serum biomarker negative at initial diagnosis or become negative during follow-up, respectively. In addition, false positive findings can be obtained, in case of B-HCG due to other malignancies, chemotherapy or marijuana use and in case of AFP due to chronic liver disease, liver damage or liver cancer [11, 12]. Therefore, there is a clinical need for biomarkers with a better sensitivity, especially related to (pure) SE and EC, for primary tumor or metastasis detection (reviewed in [13]). The advent of techniques such as microRNA (miR) profiling has allowed the identification a number of small non-coding RNAs specifically expressed in (T)GCC [14, 15]. In particular, it has been found that the expression of miR371a-3p, miR-372-3p, miR-373-3p and the miR-302a-d/367-3p cluster is characteristic for (T)GCC as well as embryonic tissues, excluding fully somatic differentiated (TE) tissues. These miRs have been reported to serve as circulating molecular serum biomarkers in patients with testicular and intracranial GCC [14–20]. It has been found that miR-371a-3p outperforms the other miRs in the detection of primary GCCs, the reason of which is still unexplained [18, 19, 21]. So far, most studies have focused on the value of these biomarkers in primary diagnosis and during chemotherapy [22], but not yet extensively during follow-up. In the current proof-of-concept study, we examined the levels of miR-371a-3p, miR-373-3p and miR-367-3p in serum samples of a follow-up series of selected TGCC patients ranging from the time of primary diagnosis to relapse and complete remission. Our results confirm the power of elevated serum miR-371a-3p, 373-3p and 367-3p levels for primary TGCC diagnosis and show the efficacy of the ampTSmiR test for TGCT relapse detection, outperforming conventional serum biomarkers.

## Materials and methods

### Clinical samples

The study was approved by the institution’s Medical Ethical Committee (MEC 02· 981 and CCR2041). We adhere to the “Code for Proper Secondary Use of Human Tissue in The Netherlands” developed by the Dutch Federation of Medical Scientific Societies (FMWV, version 2002, update 2011). Serum samples were collected between January 2000 and December 2008 in the Daniel den Hoed Cancer Center, Erasmus MC and stored in liquid nitrogen or at −80 °C until use. The patients were selected based on the number of available serum samples, as well as on the availability of clinical follow-up data. Specifically, the presence of relapse and/or residual disease was used for selection. Based on these criteria, we included serum samples of one SE patient (stage I) and five NS patients (one stage I; one stage I with a second primary tumor stage IIA and three with stage IV disease). The median age at primary diagnosis was 23.5 years, mean 26.8 (range 21–39). The sera were visually inspected for hemolysis. One sample showed moderate and 5 samples showed mild hemolysis. No samples were excluded for analysis. Further clinical details are listed in Supplementary Table 1.

### miRNA purification, quantitative real-time PCR (RT-qPCR) and interpretation

For the ampTSmiR test, miRNAs (miRs) were isolated from 50 μl serum using target specific anti-miR magnetic beads as reported before [18]. In short, a MagMax™ Express-96 robot with TaqMan® miRNA ABC Purification Kits panel A was used to isolate miRs. A non-human spike-in ath-miR-159a was added to the sera (0.2 μl of a 1 nM stock solution) for quality control of targeted miR isolation and cDNA generation. In each cDNA synthesis experiment, four ten-fold dilution series of purified miR of the TCam-2 seminoma cell line was included for quality control, qPCR efficiency and inter-plate calibration. Purified miR was reverse transcribed to miR specific cDNA using a TAQMAN MICRORNA RT KIT. cDNA generation and quantification of miR levels were performed using TaqMan Micro RNA assays for the analysis of hsa-miR-371a-3p (002124), hsa-miR-373-3p (000561), hsa-miR-367-3p (000555), ath-miR-159a (000338) and hsa-miR-30b-5p (000602). To increase sensitivity and specificity, a 13-cycle pre-amplification step was included. miR levels were determined on a TaqMan 7500 Real-Time PCR system (all apparatuses and kits: Thermo Fisher Scientific, Bleiswijk, the Netherlands). For normalization, endogenous reference miR miR-30b-5p was used [14, 18, 22]. To determine whether a sample is positive or negative, a threshold was calculated using previously generated data [18]. This set included both proven diseased (TGCC) as well as healthy males. Thresholds for positivity were calculated using the Youden index. This resulted in a 89% sensitivity and a 90% specificity for miR-371a-3p, a 70% sensitivity and a 89% specificity for miR-373-3p and a 79% sensitivity and a 85% specificity for miR-367-3p. miR-30b levels and the TCam-2 dilution series were used to compare and normalize the data generated. Data are presented as 2 ^(highest dCt in the follow-up series minus dCT)^. The values are relative, but linear and high values represent high levels.

## Results

To assess the value of the ampTSmiR test for the follow-up of TGCT patients, the miR-371a-3p, miR-373-3p and miR-367-3p levels were measured in all available serum samples of the selected patients. For each time point the standard clinical parameters were available, including the serum biomarkers B-HCG, AFP and LDH (Supplementary Table 1). The individual cases are presented one by one hereunder. The illustrations contain results of the informative serum protein and miR biomarkers only.

### Case 1

**Fig. 1:**
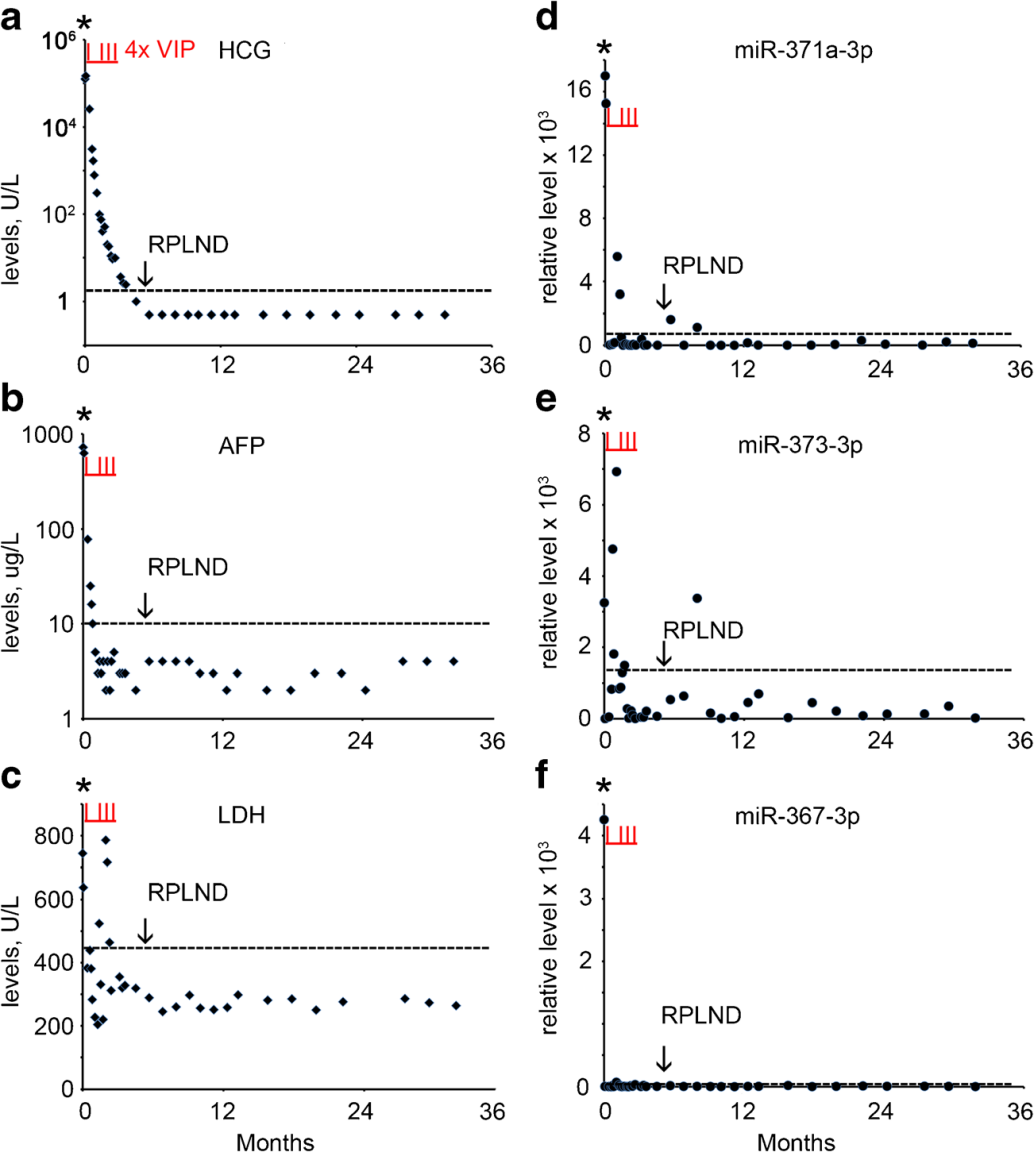
Levels of serum markers at the time of diagnosis, treatment and follow-up of TGCC case 1. **a** The levels of B-HCG (U/L), **b** AFP (μg/L), **c** LDH (U/L), **d** miRs-371a-3p, **e** 373-3p and **f** 367-3p (relative levels), during diagnosis at day 0, the time of orchidectomy (indicated by an asterisk), follow-up and RPLND retroperitoneal lymph node dissection (indicated by an arrow) are presented. (--- cut-off level for the different markers). Data are presented as 2 ^(highest dCt in the follow-up series minus dCT)^. The time of chemotherapy with vinblastine, ifosfamide and cisplatin (VIP) is marked by vertical underlined red lines (I)

A 34 year old man presented with a mass in the right testis. The conventional biomarker tests showed elevated levels of B-HCG (124,603 U/L) (normal range, < 2 U/L), AFP (722 U/L) (normal range, 0–10 U/L) and LDH (744 U/L) (normal range < 450 U/L) (Supplementary Table 1, Fig. 1a-c). The miR-371a-3p, miR-373-3p and miR-367-3p levels were also found to be elevated at primary diagnosis (Supplementary Table 1, Fig. 1d-f). The patient underwent orchidectomy of the affected testis. Histologically the tumor was composed of a mixed non-seminoma with EC, YCT, CH, SE, and immature and mature TE (ITE and MTE). CT imaging revealed enlarged mediastinal lymph nodes (MLN), left supraclavicular lymph nodes (LSLN), retroperitoneal lymph nodes (RPLN) and lung metastases, based on which the patient was diagnosed with NS stage IV, poor prognosis. Therefore, he was further treated by initially one cycle of chemotherapy with vinblastine, ifosfamide and cisplatin (VIP), followed by stem cell collection and three additional cycles of VIP and autologous stem cell treatment. Five months after the primary diagnosis a residual retroperitoneal lymph node was dissected (RPLND). None of the conventional markers and none of the miRs showed an elevated level prior to dissection, (Supplementary Table 1, Fig. 1a-f). Histopathological examination of the RPLN metastasis after dissection showed large areas of necrosis and a MTE component. Based on these observations, the patient received no further treatment and, so far, the follow-up is without any relapse, consistent with the non-elevated levels of the conventional and miR biomarkers. A single elevated level of miR-373-3p was observed 8 months after diagnosis of which the impact is still unclear. In conclusion, none of the conventional and miR biomarkers was able to detect the RPLN composed of TE only.

### Case 2

**Fig. 2:**
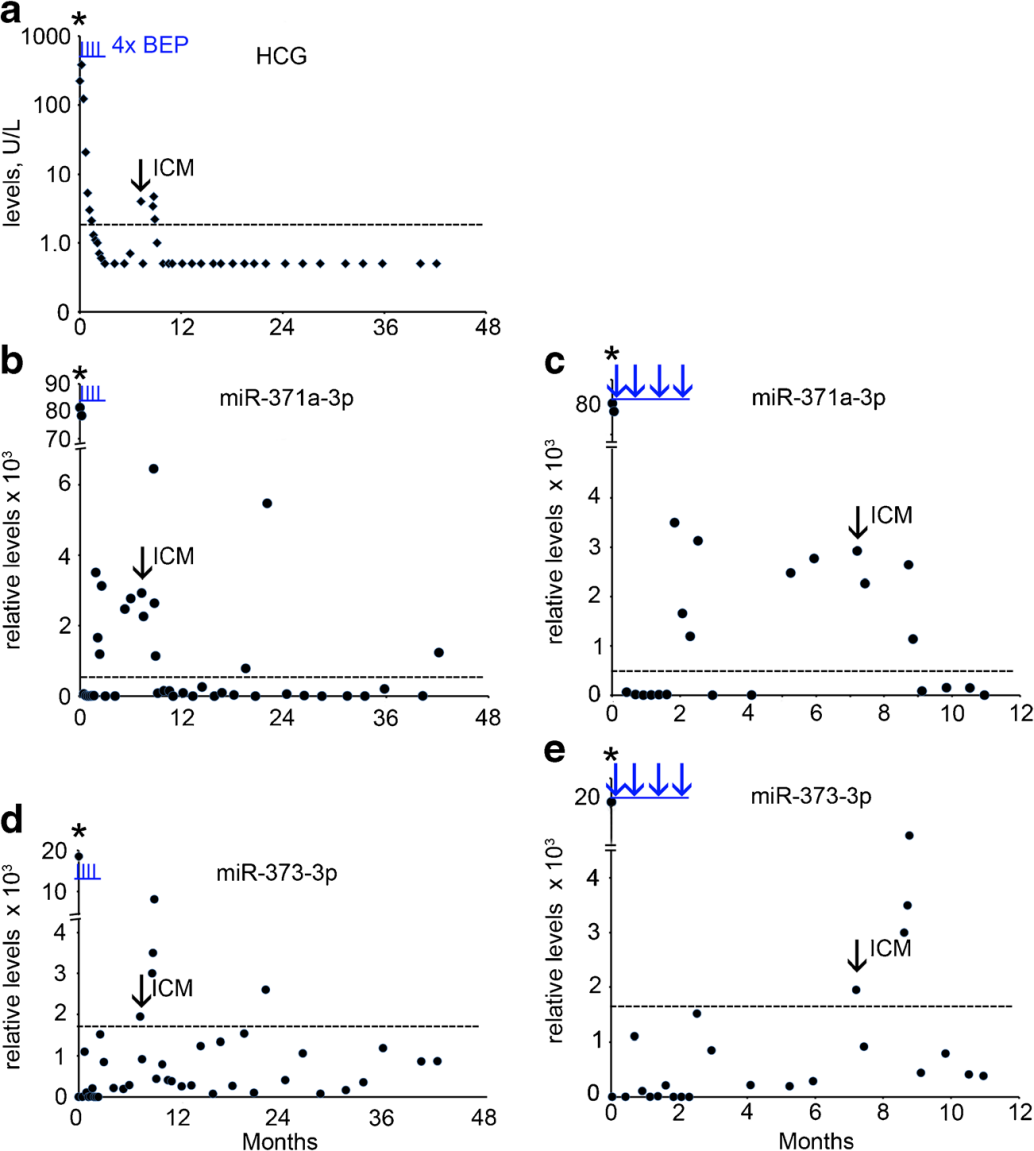
Levels of serum markers at time of diagnosis, of treatment and follow-up of TGCC case 2. **a** The levels of B-HCG (U/L), **b** miR-371a-3p, **c** miR-371a-3p during the first 12 months of follow-up, **d** miR-373-3p, **e** miR-373-3p during the first 12 months of follow-up. The time of orchidectomy is indicated by an asterisk. miRs are indicated as relative levels. Relapse at 7.2 months is indicated by an arrow. ICM; intracranial metastasis. (--- cut-off level for the different markers). The time of chemotherapy with bleomycin, etoposide and cisplatin (BEP) is indicated by vertical underlined *blue lines* (I) or *arrows* (**c** and **e**)

A 39 old man with a history of orchidopexy at the age of 14 years presented with a mass in his left testis. Conventional biomarker tests showed elevated levels of B-HCG (226 U/L) (Fig. 2a, Supplementary Table 1), AFP (19 U/L) and LDH (2441 U/L) (not shown). The ampTSmiR test revealed elevated levels of miR-371a-3p, miR-373-3p and miR-367-3p (Supplementary Table 1, Fig. 2b-e and not shown). The patient underwent orchidectomy of the affected testis, and histopathological examination revealed a mixed NS composed of EC and YST. At the moment of diagnosis CT imaging revealed the presence of enlarged inguinal lymph nodes (left) (ILN), RPLN and lung metastases. The patient was therefore diagnosed as NS stage IV, intermediate risk group. Further treatment consisted of four cycles of bleomycin, etoposide and cisplatin (BEP), followed by RPLND and inguinal LND (left sided). Histopathological examination of the lymph nodes revealed necrosis and reactive changes, without viable tumor cells. The B-HCG levels were found to decrease gradually during the first two months after surgery and chemotherapy, whereas the AFP, LDH and individual miR levels declined rapidly during the first week after the start of therapy. After 2 months, the miR-371a-3p level increased and declined again (Fig. 2b). Seven months after diagnosis the patient developed dysphasia, paresis of the right arm and seizures. MRI of the brain showed a solitary intracranial metastasis (ICM) located in the left parietal lobe. Following surgical resection, histopathological examination revealed a pure EC. The level of B-HCG was found to be moderately elevated (4.0 U/L) (Supplementary Table 1, Fig. 2a, arrow), whereas the AFP and LDH levels were normal (Supplementary Table 1). The miR-371a-3p and miR-373-3p levels were both found to be elevated at the moment of ICM detection. The miR-371a-3p levels were increased at least two months before the brain metastasis was identified (Fig. 2b, c). In contrast to the serum protein biomarkers, the miR-371a-3p levels indicated residual disease even before, and possibly related to, the development of the ICM. After surgery, treatment consisting of radiotherapy of the whole brain and a local stereotactic boost resulted in a complete clinical remission. Remarkably, for as yet unknown reasons, positive ampTSmiR scores were found for miR-371a-3p, miR-373-3p and miR-367-3p (not shown) at 15 months after relapse. The patient was lost in follow-up.

### Case 3

**Fig. 3:**
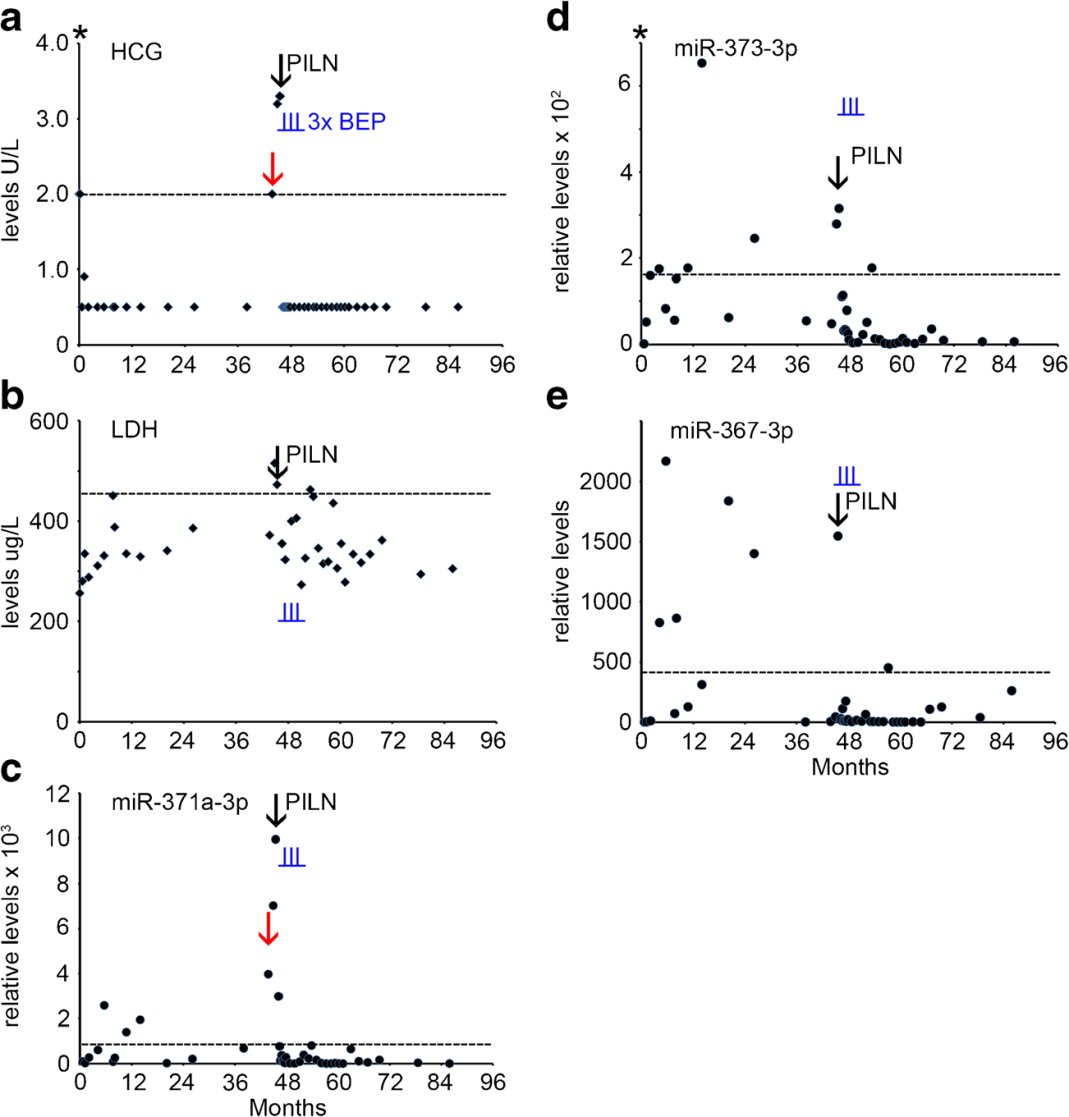
Levels of serum markers at time of start of diagnosis of TGCC case 3. **a** The levels of B-HCG (U/L), **b** LDH (U/L), **c** miRs-371a-3p, **d** 373-3p and **e** miR-367-3p (relative levels), at diagnosis, time of orchidectomy (indicated by an asterisk), and during follow-up of this series, and early relapse detection (*red arrow*) are presented. miRs are indicated as relative levels. 2^e^ p.t.; second primary tumor, PILN; para iliac lymph node, indicated by a *black arrow*. (--- cut-off level for the different markers). The time of chemotherapy with BEP is indicated by vertical underlined *blue lines* (I)

A 21 year old man underwent orchidectomy of the left testis. Histopathological and clinical examination revealed a pure SE, stage I. The level of B-HCG at diagnosis was 2.0 U/L with normal levels of AFP and LDH (Supplementary Table 1, Fig. 3, AFP data not shown). No serum sample was available for retrospective miR analysis at the time of primary diagnosis in this case. The patient was treatead prophylactically using radiotherapy of the para-aortic lymph nodes (PAOLN). After 45 months the B-HCG level increased to 2.0 U/L (Fig. 3a), with none-elevated levels of AFP and LDH (not shown and Fig. 3b). At this time point, an elevated level of miR-371a-3p was detected (Fig. 3c, red arrow), with undetectable miR-373-3p and miR-367-3p levels (Fig. 3d and e). Seven weeks later a para-iliac lymph node (PILN) metastasis was detected by CT imaging. At this time, B-HCG was modestly elevated (3.3 U/L) as well as the LDH level (473 U/L), but the AFP level was normal. Concomitantly, all the miR levels were elevated (Supplementary Table 1, Fig. 3c-e, black arrow). Because of the relapse, the patient was treated with three cycles of BEP. Both the protein and the miR biomarker levels showed a rapid drop after the start of therapy. A complete remission was achieved with a total uneventful follow-up of 42 months. The patient is still under surveillance. The levels of both B-HCG and miR-371a-3p, miR-373-3p and miR-367 indicated the presence of a PILN, of which the first miR (as well as B-HCG, being borderline) were the first to be positive. Of interest, at multiple time points positive miR levels were detected, i.e., absence of reaching an undetectable level, before the moment of PILN diagnosis. During follow-up an additional positivity was noted for miR-371a-3p, miR-373-3p and LDH, all just above the threshold levels, at the same time point.

### Case 4

**Fig. 4:**
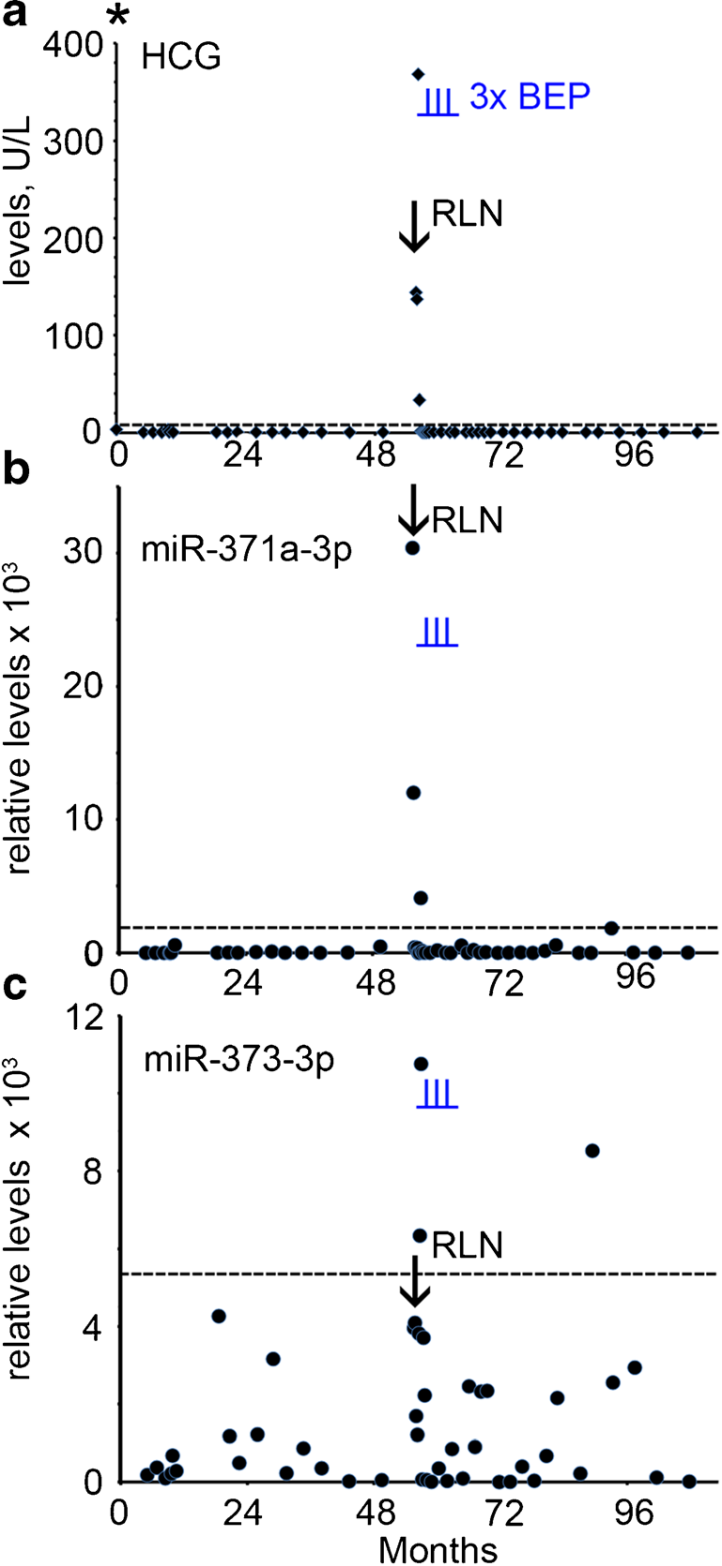
Levels of serum markers at time of diagnosis and follow-up of TGCC case 4. **a** The levels of B-HCG (U/L) at diagnosis and follow-up, **b** miRs-371a-3p, **c** 373-3p (relative levels), during follow-up and relapse are presented. The time of orchidectomy is indicated by an asterisk. The time of chemotherapy with BEP is indicated by vertical underlined *blue lines* (I). RPLN; retroperitoneal lymph node, indicated by an arrow. (--- cut-off level for the different markers)

A 25 year old man with a history of orchidopexy was treated for NS stage I by orchidectomy of the right testis. Histopathological examination revealed a pure EC. At primary diagnosis the B-HCG level was increased (3.4 U/L, Fig. 4a) with a normal AFP level and an unknown LDH level (Supplementary Table 1, and not shown). No serum was available for retrospective miR analysis at this time point. The patient was followed-up by active surveillance. Four years after diagnosis the B-HCG level was found to be increased (144 U/L) (Fig. 4a) and a retroperitoneal lymph node metastasis was detected by CT imaging. Retrospective analysis showed that the miR-371a-3p level was elevated at the time of relapse (Supplementary Table 1 and Fig. 4b, arrow). The miR-373-3p level was below cut-off level (Fig. 4c). The patient was treated with three cycles of BEP and a complete remission was achieved with a total follow-up of nine years. A single positive score for miR-373-3p and miR-371a-3p (just above cut-off level) was obtained seven years after primary diagnosis, but without known clinical consequences.

### Case 5

**Fig. 5:**
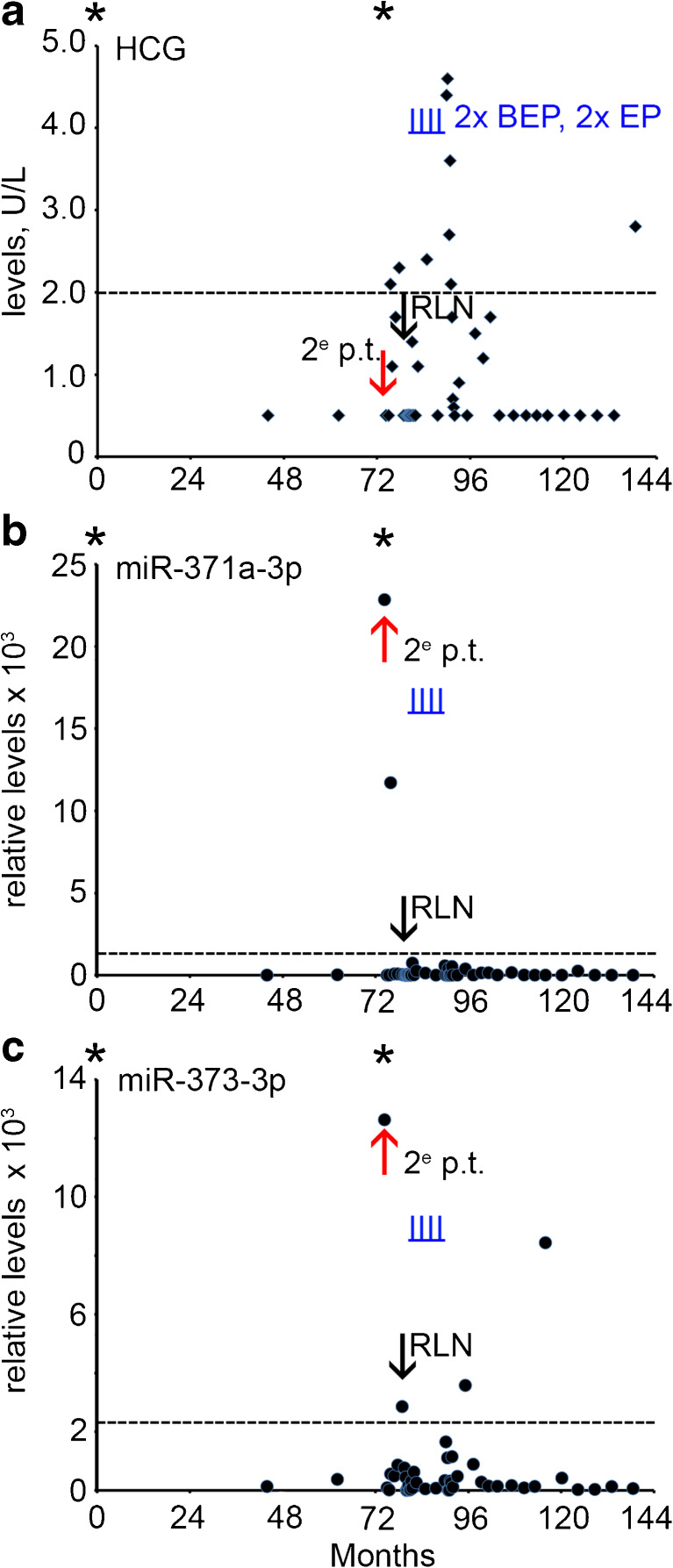
Levels of serum markers at time of diagnosis, treatment and follow-up of case 5 with a secondary TGCC (*red arrow*). **a** The levels of B-HCG (U/L), **b** miRs-371a-3p, **c** 373-3p (relative levels), during follow-up of this series and relapse (5 months after second orchidectomy, indicated by a *black arrow*) are presented. The time of orchidectomy is indicated by an asterisk. 2^e^ p.t.; second primary tumor, RPLN; retroperitoneal lymph node. (--- cut-off level for the different markers). Time of chemotherapy with BEP and EP is indicated by vertical underlined *blue lines* (I)

A 20 year old man presented with a mass of the right testis. The patient was diagnosed and treated for SE stage I by orchidectomy and radiotherapy of the PAOLN followed by active surveillance. After six years a second primary tumor was detected in the left testis (30 months after start of this follow-up series). Histopathological and clinical examination showed that it was a NS with SE and EC components, stage IIA. The levels of the conventional biomarkers were normal (Supplementary Table 1). The miR-371a-3p and miR-373-3p levels were, however, found to be increased (Fig. 5b and c, red arrow). The patient was treated with 2 cycles of BEP and 2 cycles EP. Five months after the second orchidectomy a RPLN metastasis was detected by CT imaging. The levels of B-HCG, AFP and LDH were not increased at this time point (Fig. 5a and not shown), whereas of the different miRs tested miR-373-3p showed an elevated level just above the threshold (Fig. 5c, black arrow). During follow-up two serum samples were found to be miR-373-3p positive, without known clinical consequences. As yet, the impact of these observations remains unclear, but the patient is still under surveillance. The patient was treated with two cycles of BEP and two cycles of EP. Of notice, the levels of B-HCG were often found to be increased during follow-up (Fig. 5a), probably due to increased levels of follicle-stimulating hormone caused by the bilateral orchidectomy [23]. Such a pattern was not observed for the miRs.

### Case 6

**Fig. 6:**
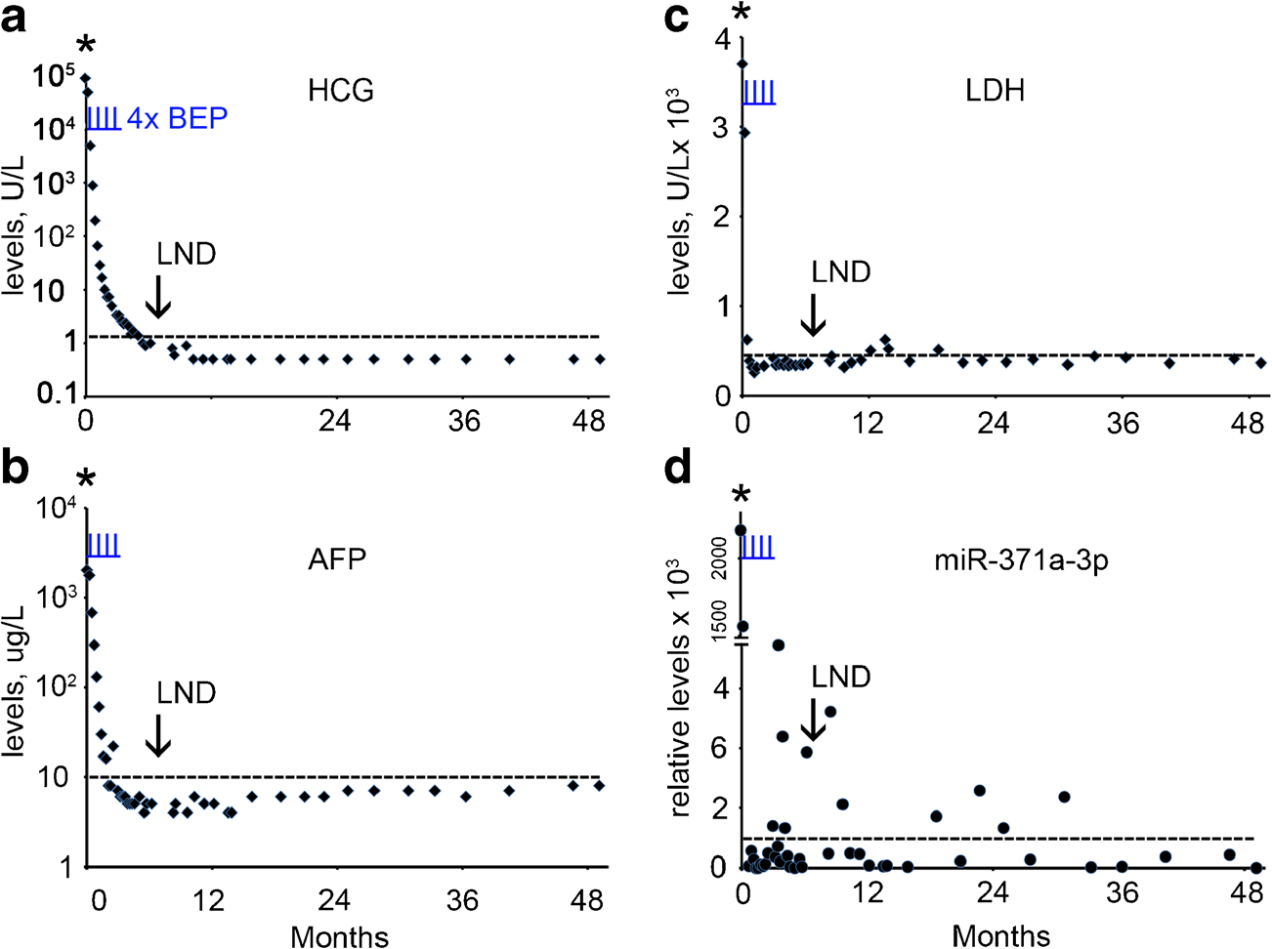
Levels of serum markers at time of diagnosis, treatment and follow-up of TGCC case 6. **a** The levels of B-HCG (U/L), **b** AFP (μg/L), **c** LDH (U/L), **d** miRs-371a-3p (relative levels), during diagnosis, follow-up and relapse are presented. The time of orchidectomy is indicated by an asterisk. (--- cut-off level for the different markers). The time of chemotherapy with BEP is indicated by vertical underlined *blue lines* (I). LND; lymph node dissections indicated by an *arrow*

A 22 year old man with a history of orchidopexy of the right testis at age of 11 years presented with a mass in his left testis. The conventional biomarker tests showed highly elevated levels of B-HCG (88,915 U/L), AFP (2024 U/L) and LDH (3698 U/L) (Fig. 6a-c). Similarly, miR-371a-3p, miR-373-3p and miR-367-3p showed high levels (Supplementary Table 1, Fig. 6d and not shown). The patient underwent orchidectomy of the affected testis. Histopathological examination showed that the testis consisted of MTE and necrosis components (NS, MTE). CT imaging revealed lung and liver metastases, and enlarged PAOLN. The patient was, therefore, diagnosed as NS stage IV with a poor prognosis. The treatment consisted of four cycles of BEP. After seven months a residual bilateral RPLND and inguinal LND were performed, as well as orchidectomy of the right testis. Histopathological examination showed that the RPLN, ILN and testis were all composed of pure MTE. The levels of B-HCG, AFP and LDH were found to be normal (Supplementary Table 1, Fig. 6a-c, black arrow). Based on the clinical data and the levels of the conventional biomarkers, no further chemotherapy was decided. However, the patient experienced a late relapse after seven years of follow-up, consisting of lung and RPLN metastases. Histopathological examination of the LND showed that it was a NS composed of CH only. The serum biomarkers were normal and, unfortunately, no serum was preserved for retrospective miR analysis. The therapy consisted of three cycles of paclitaxel, ifosfamide and cisplatin (TIP) and radiotherapy, but no complete remission was achieved. Retrospective revision of the RPLND at seven months of follow-up indicated that a CH component was overlooked. In line with this finding, retrospective analysis showed that the miR-371a-3p level was elevated in four serum samples collected before the operation (Fig. 6d), providing a strong indication for the presence of other components than MTE alone.

## Discussion

Years or even decades after their initial diagnosis, (T)GCC patients have a risk to develop a second TGCC or a recurrence of the disease. Therefore, they require intensive and long term follow-up after diagnosis and treatment [24]. Follow-up strategies differ between centers, but include anamnesis, physical examination, evaluation of serum tumor markers and repeated radiological imaging. The basic question addressed in this proof-of-concept study is whether embryonic miR levels can predict a relapse of (T)GCC better than the conventional tumor biomarkers B-HCG, AFP or LDH. Although the outcomes of this study must be validated in, currently ongoing, independent larger cohorts of patients, the results indicate that the ampTSmiR test outperforms the conventional biomarkers in detecting both a primary tumor, residual disease and/or relapse. For as yet unknown reasons, a few positive results were observed at time points without recorded clinical events. This might be related to the sensitivity and specificity thresholds set for this study. These were based on a previous study in which proven diseased (TGCC) as well as healthy males were compared [18]. In a clinical setting, a positive miR finding should therefore be repeated after a week, as is the standard for conventional serum markers. As of yet, miR analyses should be performed in addition to conventional serum biomarker analyses but, in addition to that, they could already be very valuable for conventional biomarker negative patients.

In the current series of 6 patients, 5 were found to be positive based on conventional serum biomarker assays at the time of primary diagnosis. The actual frequency is, however, lower in the general population of (T)GCC patients (~60%) [25], thereby complicating follow-up. Although a relatively small number of patients is analyzed, a number of conclusions can be drawn. The miR scores were positive in all four available cases at primary diagnosis, which is in agreement with the sensitivity of the ampTSmiR test published before [18, 26], thereby substantiating the added value of miR profiling at primary diagnosis. Three out of four cases with a relapse were B-HCG positive and one case was LDH positive. One case tested negative for all protein serum biomarkers. The sera of all four cases were at least positive for one miR, indicating the possible additional value of the ampTSmiR test for the follow-up of TGCC patients. Of the three selected miRs, miR-371a-3p was found to be the most sensitive and specific one and to outperform the others in detecting a relapse, which is in agreement with recently published results of larger series of patients with a shorter follow-up and less measure points [19].

Our series includes two patients (no. 1 and 6) with a residual RPLN after chemotherapy composed of pure MTE. Both cases showed a normal level of the conventional serum biomarkers, as expected based on the fact that pure TEs do not secrete HCG or AFP and do not express embryonic miRs [18, 27]. The failure to detect pure TE with conventional serum biomarkers and the ampTSmiR test is of clinical importance since this chemo-resistant entity requires surgery as treatment to prevent further progression towards non-germ cell malignancy. Retrospective analysis of available serum samples revealed that case no. 6 exhibited increased miR-371a-3p levels in the period before RPLND, which is indicative for the presence of other components than TE alone. Indeed, as mentioned above, histopathological revision of the pathological samples of this case revealed that the biopsy material contained CH, which was overlooked at earlier diagnosis. Both observations may be indicative for additional (chemo)therapy.

The diagnosis of an intracranial germ cell tumor is based on clinical symptoms, the detection of biomarkers such as AFP and B-HCG in blood and cerebrospinal fluid, magnetic resonance imaging of the brain and spinal cord, CSF cytology and histology. Here we show that, besides B-HCG, miR-371a-3p and miR-373-3p are able to predict the presence of an ICM as a TGCT relapse. This notion indicates that the miRs can pass the blood brain barrier (BBB) and enter the peripheral blood circulation, in line with published data [28]. In our case no. 2 the miR-371a-3p levels were high during VIP therapy, decreased for a month after therapy and increased again two months before serum protein biomarker positivity and the detection of ICM, strongly indicating that no complete remission was achieved.

## Conclusions

From our data we conclude that the ampTSmiR test is highly sensitive and specific for the identification of TGCC patients at primary diagnosis, as well as residual disease and relapse. The results of this proof-of-concept study warrant further evaluation of this molecular biomarker in a larger cohort of patients with possible active disease during follow-up.

(AFP), α-fetoprotein; (BEP), bleomycin, etoposide and cisplatin; (B-HCG), human chorionic gonadotropin subunit beta; (CH), choriocarcinoma; (EC), embryonal carcinoma; (ICM), intracranial metastasis; (LDH), lactate dehydrogenase; (miRs), microRNAs; (NS), non-seminoma; (RPLN), retroperitoneal lymph node; (RPLND), retroperitoneal lymph node dissection; (TE), teratoma; (TGCC), testicular germ cell cancer; (VIP), vinblastine, ifosfamide and cisplatin; (YST), yolk sac tumor.

## Electronic supplementary material

ESM 1 (DOCX 20 kb)
