# Supplementary material for: microRNA-371a-3p as informative biomarker for the follow-up of testicular germ cell cancer patients
Source: Cell Oncol (Dordr). 2017 Jun 13;40(4):379–88. doi: 10.1007/s13402-017-0333-9 (PMC5537315; doi:10.1007/s13402-017-0333-9)
Supplement: Supplementary file 1 — (DOCX 20 kb) [file 13402_2017_333_MOESM1_ESM.docx]

Appendix Table I. Clinicopathologic factors

| **Case** | **1** | **2** | **3** | **4** | **5** | **6** |
| --- | --- | --- | --- | --- | --- | --- |
| **Diagnosis** |  |  |  |  |  |  |
| **Orchidopexy (age)** | No | 14, left | No | Yes | No | 11, right |
| **TGCC (age)** | 34 | 39 | 21 | 25 | 20*, 26** | 22 |
| **Histology** | NS: EC, YST, CH, SE, ITE, MTE | NS: EC,YST | SE | NS: EC | SE*  NS**: SE, EC | NS, MTE |
| **Stage and metastasis** | IV: MLN, SLN , RLN, lung meta | IV, ILN, RLN, lung meta | I | I | I*, IIA** | IV: lung, liver, PAOLN |
| **Serum markers** |  |  |  |  |  |  |
| B-HCG (U/L) | **12,4603** | **226** | 2 | **3.4** | 0.5* | **88915** |
| AFP (µg/L) | **722** | **19** | 2 | 3.3 | 1* | **2024** |
| LDH (U/L) | **744** | **2,441** | 256 | n.a. | 399* | **3698** |
| **Serum miRNA** |  |  |  |  |  |  |
| miR-371a-3p | **Pos** | **Pos** | n.a. | n.a. | **Pos*** | **Pos** |
| miR-373-3p | **Pos** | **Pos** | n.a. | n.a. | **Pos*** | **Pos** |
| miR-367-3p | **Pos** | **Pos** | n.a. | n.a. | Neg* | **Pos** |
| **Surgery** | orchiectomy, right | orchiectomy, left | orchiectomy, left | orchiectomy, right | orchiectomy,  right*, left** | orchiectomy, left |
| **Treatment** | 1x VIP, | 4x BEP | RT, PAOLN |  | RT,PAOLN*,2xBEP,2xEP** | 4x BEP |
|  | 3x VIP, stem cell treatment |  |  |  |  |  |
| **Follow-up (months)** | 32 | 30 | 86 | 106 | 127 | 49 |
| **Event (months)** | 5 | 7 | 46 | 44 | 5 | 6 |
| **Diagnosis** | RLN | ICM | PILN | RLN | RLN** | RLN |
| **Histology** | MTE | EC | n.a. | n.a. | n.a. | MTE, CH |
| **Treatment** | RLND | RT | 3x BEP | 3x BEP | 2x BEP, 2 EP** | RLND, ILND, orch. |
| **Serum markers** |  |  |  |  |  |  |
| B-HCG (U/L) | 1 | **4.0** | **3.2** | **144** | 0.5** | 1 |
| AFP (µg/L) | 2 | 2 | 2 | 3 | 1** | 5 |
| LDH (U/L) | 319 | 330 | **473** | 300 | 302** | 363 |
| **Serum miRNA** |  |  |  |  |  |  |
| miR-371a-3p | Neg | **Pos** | **Pos** | **Pos** | Neg** | **Pos** |
| miR-373-3p | Neg | **Pos** | **Pos** | Neg | **Pos**** | Neg |
| miR-367-3p | Neg | neg | **Pos** | Neg | Neg** | Neg |
| **Main outcome** | CR | CR | CR | CR | CR** | late relapse |

Abbreviations: TGCC; testicular germ cell cancer, NS; non-seminoma, EC; embryonal carcinoma, YST; yolk sac tumor, CH; chorio carcinoma, SE; seminoma, ITE; immature teratoma, MTE; mature teratoma, MLN; mediastinal lymph node, SLN; supraclavicular lymph node, RLN; retroperitoneal lymph node, n.a.; serum or tissue not available, VIP; vinblastine, ifosfamide and cisplatin, BEP; bleomycin, etoposide and cisplatin, RT; radio therapy, PAOLN; para aortal lymph node, ICM; intracranial metastasis, PILN; para iliac lymph node, ILN; inguinal lymph node, CR; chronic remission, * first primary tumor, **; second primary tumor. Increased values above thresholds are in bold depicted.
